# LOPOSTER: A Cascading Postprocessor for LOBSTER

**DOI:** 10.1002/jcc.70167

**Published:** 2025-06-30

**Authors:** YiXu Wang, Peter C. Müller, David Hemker, Richard Dronskowski

**Affiliations:** ^1^ Chair of Solid‐State and Quantum Chemistry Institute of Inorganic Chemistry, RWTH Aachen University Aachen Germany

**Keywords:** carbodiimide, chemical bonding, LOBSTER, magnetic ordering, postprocessing

## Abstract

The computer program LOPOSTER, available via GitHub, is introduced, capable of postprocessing the LOBSTER code results. LOPOSTER is designed to be particularly effective for analyzing large datasets with over 10,000 interactions and enormously reducing postprocessing time. LOPOSTER pioneers the automated processing of advanced bonding analysis results, including multicenter bonding, molecular‐orbital formation energy, and *k*‐dependent COHP, expanding the scope of routine chemical‐bonding investigations. In addition, LOPOSTER streamlines the postprocessing workflow by providing comprehensive results in a single execution, minimizing user intervention and potential errors. An example of chemical‐bonding analysis on NiNCN is provided, with visualization by LOPOSTER. LOPOSTER offers versatile analysis of interactions in NiNCN, enabling evaluations in real or reciprocal space, and based on atomic or molecular orbitals, catering to different analytic preferences. Various correlations between those interactions and magnetism in NiNCN are also explored. The electron‐rich features of an N=C=N π bond have been discussed from various perspectives.

## Graphical abstract

LOPOSTER is a robust and highly efficient algorithm for fully automated postprocessing of chemical bonding analysis as produced by LOBSTER.

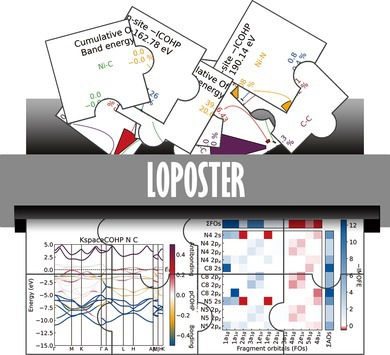

## 1 Introduction

Chemical bonding is one of the most essential notions in chemistry, actually the core of chemistry, and an excellent metaphor for interactions between atoms [1]. In a nutshell, the concept of chemical bonding encompasses mainly two aspects: covalency and ionicity. Covalency originates from quantum‐mechanical superposition and (constructive and destructive) interference of atomic wavefunctions. In contrast, ionicity is rooted in classical Coulomb electrostatic interactions (even though also from interference of wave functions energetically far apart). Metallicity is treated as an extreme case of covalency tackling too few valence electrons in a system with a plethora of atoms. This means that quantitative analysis of chemical bonding offers insights into the structure–property relationships in the given system, be it a molecule or a solid.

Computational chemistry plays an increasingly vital role in quantitatively analyzing and understanding chemical bonding in complex systems. In recent years, various methodologies have been developed to perform such analyses, and they can be broadly classified into two categories: electron‐density based and wavefunction‐based methods. Both methods typically rely on highly accurate *ab initio* calculations, commonly performed by density‐functional theory (DFT). Electron‐density information is readily accessible and directly relates to experimental observables. Wavefunction‐based methods point, however, directly to the quantum‐mechanical nature of covalency (= interference). Furthermore, this approach is easily extendable to the analysis of bonding involving multiple atomic centers. A challenge arises from the fact that most solid‐state DFT results are performed using plane‐wave basis sets. This methodology, typically employing the mighty combination of popular projector augmented wave (PAW) pseudopotentials and plane waves in addition to, say, the generalized gradient approximation (GGA) for the exchange–correlation functional (or more expensive alternatives), can obscure the essential phase information of the wavefunction. However, the phase information of the wavefunction is of paramount importance to wavefunction‐based chemical‐bonding analysis but is inaccessible under the delocalized plane‐wave DFT methodology.

Nonetheless, a unitary reciprocal‐space transformation can be applied to the delocalized plane‐wave DFT results allowing for their interpretation in terms of localized atomic‐orbital basis sets. To address this, the computer program Local‐Orbital Basis Suite Toward Electronic‐Structure Reconstruction [2, 3, 4, 5] (LOBSTER) has been developed over the past decade. LOBSTER offers automatic solutions for such unitary transformation, enabling wavefunction‐based chemical‐bonding analysis of plane‐wave DFT results, analogous to methods used for molecular systems. LOBSTER resembles a quantum‐chemical Swiss army knife offering wavefunction‐based chemical‐bonding analytic options represented by crystal orbital Hamilton population (COHP) [6] and its analogs such as crystal orbital overlap population (COOP) [7, 8] and crystal orbital bond index (COBI) [9], partitioning/evaluating the density of states (DOS) information by Hamilton, overlap, and density matrix, respectively. Since the first release of LOBSTER, this very tool has been applied to various material systems [1] in the fields of solid‐state chemistry, condensed matter physics, and materials science and engineering.

In 2025, LOBSTER users are simulating larger and larger systems, thanks to the skyrocketing power of computational facilities. As a result, the number of chemical bonds in those systems is climbing, making the postprocessing of bonding information a potentially demanding task for researchers. The increasing size and complexity of simulated systems in modern computational chemistry necessitate efficient postprocessing tools to handle the vast amount of bonding information generated. At present, there exist a few codes that can provide postprocessing services, i.e., wxDragon [10], Multiwfn [11] and lobsterpy [12, 13]. Clearly, wxDragon and Multiwfn are more suitable for data visualization whereas lobsterpy achieves automatic postprocessing but requires installation of python environment first. A fully automatic, out‐of‐the‐box and user‐friendly standalone postprocessor for researchers with limited programming expertise, yet capable of performing all necessary postprocessing with a *single* run remains a highly desirable tool. Herein, we present the LOPOSTER code designed to perform all necessary postprocessing of LOBSTER output. Similar to the existing tools, LOPOSTER provides visualization functionalities for the datasets produced by LOBSTER and helps to generate a suitable lobsterin file to facilitate the usage of LOBSTER. Nonetheless, appending to the existing functionalities, LOPOSTER distinguishes itself by offering a fully automated, user‐friendly solution for comprehensive postprocessing of LOBSTER outputs in a single execution, addressing the limitations of existing tools in terms of automation, ease of use for non‐programmers, and comprehensive analysis capabilities. To name a few, LOPOSTER supports parallelization to increase the efficiency of data processing, and also offers functions to plot multicenter COBI, cumulative on‐site and off‐site COHP, *k*‐space COHP and the integrated molecular orbital formation energy (IMOFE) matrix. In addition, LOPOSTER assists in checking the convergence of self‐consistent field (SCF) calculations, generating lobsterin files for LOBSTER execution, raising warnings once abnormal criteria are fulfilled (see text below), automatically searching for LOBSTER output files and finishing the postprocessing job in a completely automatic way. In what follows, we give an overview of LOPOSTER and also provide an example of chemical‐bonding analysis on nickel carbodiimide, NiNCN, containing the complex [NCN]^2−^ carbodiimide anion.

## 2 Methods and Computational Details

Quantum‐mechanical calculations were carried out using the Vienna *ab initio* simulation package (VASP, version 6.1.1) [14, 15]. Projector‐augmented‐wave (PAW) pseudopotentials within the Perdew–Burke–Ernzerhof [16] (PBE) framework were used, and the generalized gradient approximation (GGA) [17] scenario was chosen to treat both exchange and correlation. Crystal structures were geometrically optimized until the energy difference and force difference between consecutive iterations were less than 10^−8^ eV and 10^−6^ eV/Å, respectively. Gaussian smearing with a width of 0.02 eV was chosen for geometry optimizations. Upon convergence, a single point calculation was performed with an energetic convergence criterion of 10^−8^ eV to generate the PAW wavefunction information. Tetrahedron method with Blöchl corrections [18] was utilized for sampling the first Brillouin zone. The energy cutoff was set at a large value of 800 eV. A 9 × 9 × 11 Γ‐centered Monkhorst–Pack *k*‐point mesh (for regular analysis) or an explicitly specified *k*‐point list with equivalent distance between two neighboring *k* points (for *k* space analysis, helped by the python package seekpath [19]) was generated to sample the first Brillouin zone. The distance between two adjacent *k* points would not exceed 2π × 0.02 Å^−1^. Three conditions were considered: 1. Calculations were performed without considering spin polarization, dubbed *nonmagnetic* (NM); 2. Calculations were conducted with spin polarization and a *ferromagnetic* initial magnetic configuration (FM); 3. Calculations were carried out with spin polarization and an *antiferromagnetic* initial magnetic configuration [20] (AFM) where magnetic moments (in NiNCN) order ferromagnetically within the *ab* plane and antiferromagnetically along the *c* axis. LOBSTER 5.1.1 was employed to process the DFT results from VASP and produce the output files to feed LOPOSTER.

**FIGURE 1:**
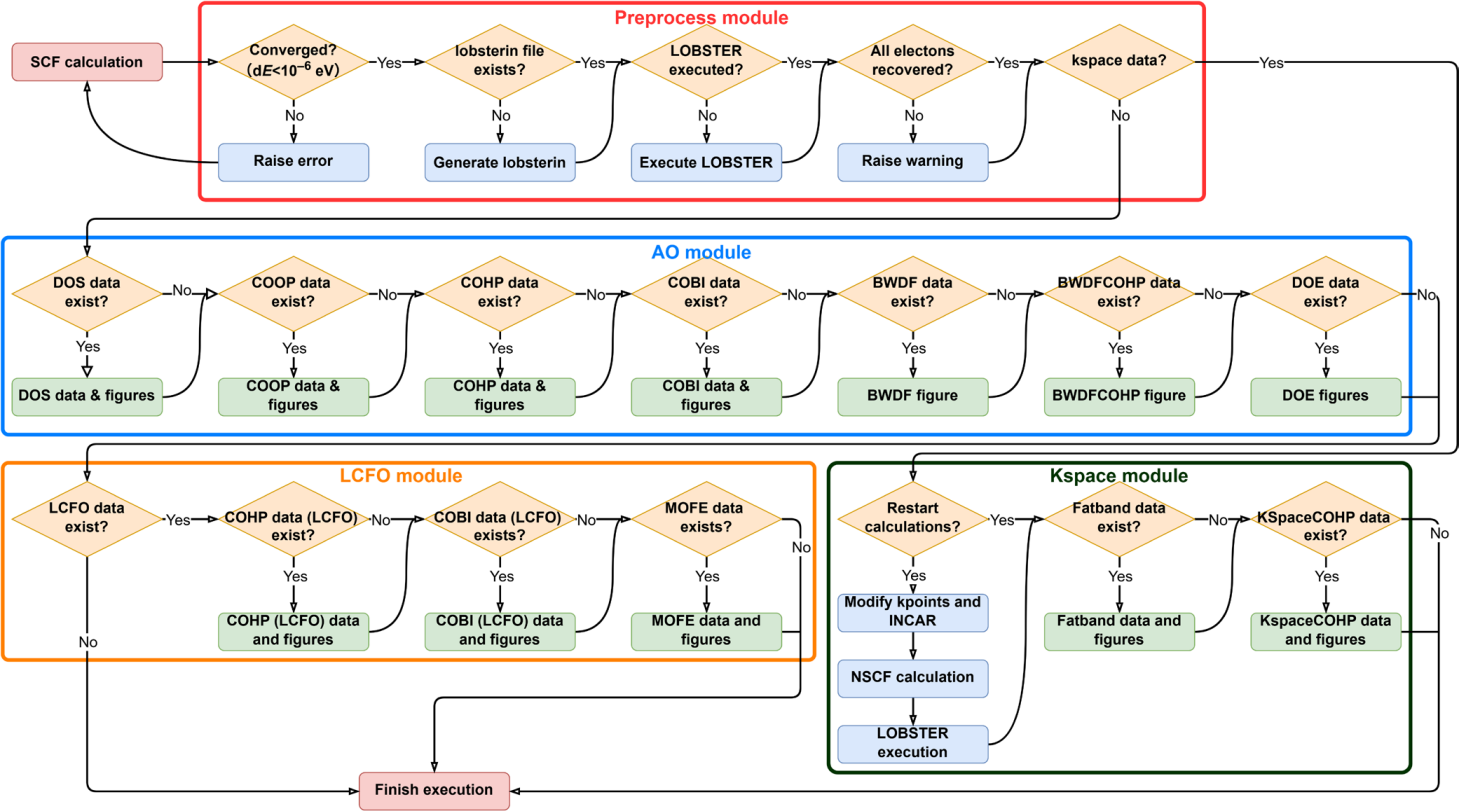
Schematic flow chart of LOPOSTER.

The LOPOSTER program was written in Python and built based on only four external dependencies: Numpy [21] for data processing, Matplotlib [22] for data visualization, seekpath [19] for generating *k* point list, and tqdm [23] for decorative progress bars. To serve the users better in terms of speed and convenience, the python source code of LOPOSTER would be packed (helped by pyinstaller) into standalone executable files for various operating systems. It is worth noting that LOPOSTER is not a script based on lobsterpy and packed by pyinstaller. The users can use LOPOSTER through a simple double‐click (in case of Windows) without any prerequisite for Python environments. The program LOPOSTER consists of four modules: preprocess module, atomic orbital (AO) module, linear combination of fragment orbital (LCFO) module, and kspace module. The basic workflow of LOPOSTER has been summarized and sketched in Figure 1. Upon invoking the program, it always starts from the preprocess mode, judging the completeness of the calculations and the type of job under the current directory. When the job is related to the calculations in real space, the postprocessing is cascaded to the AO module. Otherwise, the postprocessing is cascaded to the kspace module. An additional judgment is undertaken when the AO module finishes to see if there is any dataset from LCFO. If yes, the postprocessing is cascaded to the LCFO module. Within each module, pipelines of judgments are arranged to look for corresponding datasets for postprocessing. The preprocess module checks if the SCF calculations have been converged with energy difference below a decent threshold (10^−6^ eV), if the lobsterin file exists, if LOBSTER has been executed, if all electrons have been recovered by LOBSTER, and if the data to be fed into LOPOSTER are in real or reciprocal space. Subsequently, AO module looks for the datasets generated using atomic orbitals and performs visualizations of data in each dataset (or combination of datasets). Similarly, the LCFO module plots COHP/COBI data (with fine customizations) in datasets based on fragment orbitals. Moreover, the molecular orbital formation energy, for the first time, can also be depicted by the LCFO module (see text below). As regards the *k*‐dependent data, kspace module will automatically search for related datasets and take care of plotting fatband and *k*‐space COHP data. The prepacked executable files and examples to reproduce the results from this work can be found on GitHub under https://github.com/WangYiXu92/LOPOSTER.

## 3 Results and Discussion

### 3.1 Preprocess Module

The module got its name due to the fact that it attempts to bridge the gap *after* the SCF calculations and *before* commencing data processing in LOPOSTER using the datasets generated by LOBSTER. Such a module necessitates its existence by creating a concrete background for the subsequent chemical bonding analyses. Before proceeding with the output from LOBSTER as the input for LOPOSTER, quality checks have to be performed, guaranteeing reliable chemical bonding information extracted from plane‐wave theory.

Once the SCF calculations have been completed, the first step is to check whether convergence was reached or not since further postprocessing jobs can only be embarked on if the convergence criterion (please refer to the details in the “Methods and Computational details” section) is fulfilled. In LOPOSTER, we also need to check if the convergence criterion is tight enough. Wavefunctions obtained using a convergence threshold above 10^−6^ eV are not considered to be fully converged, ensuring an excellent starting point from plane‐wave theory to be projected via atomic orbital basis sets. Subsequently, LOPOSTER will search for a lobsterin file. If there is none, LOPOSTER will automatically *generate* a suitable lobsterin file based on the information from the structure file. The default options to token are: saveProjectionToFile, loadProjectionFromFile, COHPStartEnergy, COHPEndEnergy, autoRotate, gaussianSmearingWidth, basisSet, basisFunctions, cohpGenerator, DensityOfEnergy, BWDF, and BWDFCOHP. Specifically, default settings for COHPStartEnergy, COHPEndEnergy, gaussianSmearingWidth are −15, 5, and 0.05 eV, respectively. The default basis set is pbeVaspFit2015. As for the basis functions, LOPOSTER can automatically set them based on the valence electrons found from POTCAR and specify every basis function for the same element in the same line. Currently, this function only supports VASP. As regards the cohpGenerator, a cutoff of 4 Å is set as the upper and 0 Å as the lower bound. The keyword orbitalwise is also appended to extract as many details as possible from a single LOBSTER run. In what follows, a three‐center bond has been manually specified for the carbodiimide moiety and the same set of atoms has been manually assigned as an embedded molecule for analysis in terms of linear combination of fragment orbitals (LCFO).

After generating the lobsterin file, one needs to execute LOBSTER to produce the data files that will be pipelined to LOPOSTER. LOPOSTER also helps to detect if a LOBSTER run has been finished so that all the datasets needed for chemical bonding analysis have been written by LOBSTER. From the lobsterout file, the number of electrons recovered from projection is very important to check but, to the best of our knowledge, none of the aforementioned codes checks it. This very number of electrons is an essential parameter to evaluate the quality of a LOBSTER run. Technically, *all* electrons can be recovered [24] by LOBSTER for an (almost) complete basis set; abnormal cases of less/more recovered electrons may indicate an inappropriate choice of basis functions or/and basis set, a very rare case which needs manual intervention. At the end of the preprocess module, an important judgment of whether the calculation is a real space analysis or a *k* space analysis has been undertaken. According to the results of the judgment, different modules will be called for. For real space analysis, the job will cascade to the AO module. Otherwise, the job will proceed through the kspace module.

### 3.2 AO Module

This module will take care of the results produced using atomic‐orbital basis functions. LOPOSTER program will automatically look for the following files: DOSCAR.lobster, COOPCAR.lobster, ICOOPLIST.lobster, COHPCAR.lobster, ICOHPLIST.lobster, COBICAR.lobster, ICOBILIST.lobster, DensityofEnergy.lobster, BWDF.lobster, and BWDFCOHP.lobster. On platforms supporting multiprocessing, which is the case of modern central processing unit (CPU) architecture, plotting those files will proceed in parallel. Data from DOSCAR.lobster, DensityofEnergy.lobster, BWDF.lobster, and BWDFCOHP.lobster would be plotted into separate files with names of DOS, DOE, BWDF, and BWDFCOHP, respectively. For files with CO*x*CAR.lobster (*x* = OP, HP, or BI) general names, the data structure is rather similar within the framework of two‐center bonds. The bond information, including the pair of atoms at both ends of the bond and the distance between them, will be read and extracted from the header of those files. The integrals will be extracted from the corresponding ICO*x*LIST.lobster (*x* = OP, HP, or BI) file. Successively, the data will be grouped according to the elements and distances. Initially, bonds with the same length and the same kind of atoms/elements are defined as being identical. Data from identical bonds would be averaged to combat numerical noise and gain a more comprehensive picture of the bonding situation. In the meantime, the standard deviation of the data would also offer us one more perspective on analyzing chemical bonds in the crystal structure. After finishing the postprocessing of the datasets in the scope of AO module, LOPOSTER will proceed to look for files generated through the LCFO functions in LOBSTER.

**FIGURE 2:**
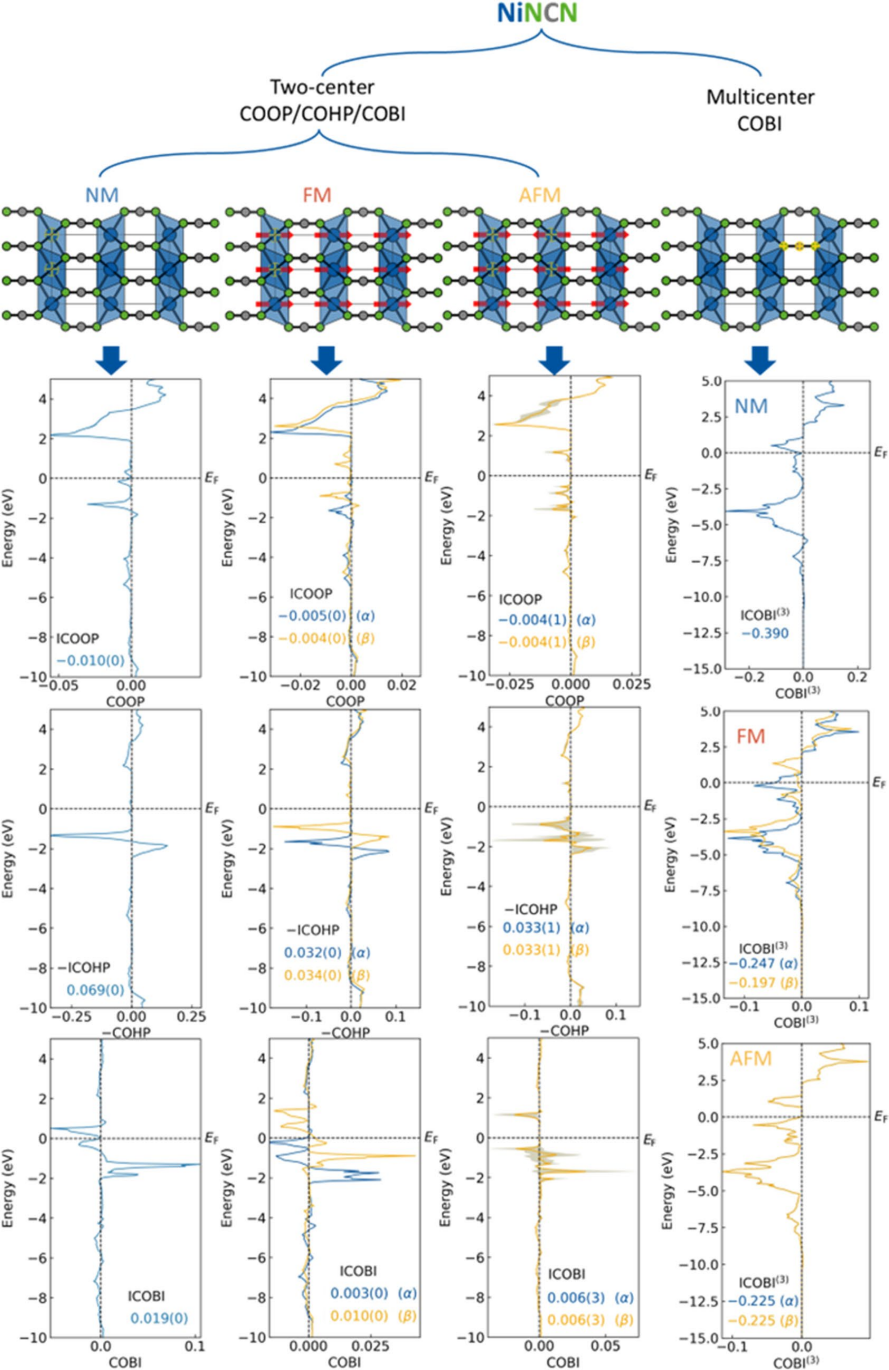
Crystal and magnetic structures of NiNCN and course of COOP, COHP and COBI analysis for the Ni–Ni bond in NM, FM, and AFM situations. Red arrows represent the direction of magnetic moments. The bond of interest is highlighted in yellow. For multicenter N–C–N bonding in the right part of the figure, magnetic moments have been omitted for clarity.

For illustration, Figure 2 provides averaged COOP, COHP and COBI data for the Ni–Ni interactions in NiNCN assuming NM, FM, and AFM models of the ground state; the AFM state is the one closest to reality [20, 25]. Considering the magnetism variant, one may break all interactions into two types, direct and indirect exchange interactions [26]. For reasons of simplicity and on purpose, we first focus on Ni–Ni (cation–cation interactions in NiNCN) even though these interactions belong to the weakest bonds in the structure, the Ni–N and C–N bonds being much stronger (see comparison later). The averaged data over the identical interactions have been plotted as solid lines and their numerical standard deviations sketched as shaded areas in their respective colors. The standard deviations—derived from the corresponding two‐center interactions datasets—are used as a figure of merit to evaluate the convergence of results and would be helpful to decide whether either an element‐resolved (data from the same combination of elements to be averaged) or an atom‐resolved (data from the same combination of atom to be averaged) scenario should be chosen. We will meet an example in case of AFM ordering in NiNCN.

For interatomic distances shorter than 4 Å, only two kinds of Ni–Ni interactions up to ≈3.1 Å (different for three configurations) are found. Notably, only bonds with non‐zero bond length will be automatically plotted by LOPOSTER, corresponding to the Ni–Ni pair within the *ab* plane. In contrast to NM and FM models where all considered Ni–Ni interactions are treated as equivalent from a crystallographic perspective, the AFM configuration breaks such equivalence because, as the consequence of a missing 6_3_ screw axis, the Ni atom at the origin is no longer treated the same as the one at *c*/2 position. Therefore, two different kinds of Ni–Ni interactions with the same bond length should be taken into account. This is also reflected by the abnormally large standard deviations in the two‐center interactions curves especially occurring in the AFM configuration, not being due to the inaccurate DFT calculations or unitary transformation but coinciding with the physics picture that the AFM ordering can be interpreted as a broken time‐reversal symmetry (𝒯) on the microscopic level whereas 𝒯 gets restored from the macroscopic view. Together with the two‐center bonding information, multicenter bonds can also be specified but this has to be accomplished manually. In the present case, one complex anionic [NCN]^2−^ entity from the crystal structure is analyzed as an N–C–N three‐center bond. LOPOSTER can automatically sort out multicenter bonds from two‐center ones. Because the multicenter bonds are manually defined, no average will be undertaken by LOPOSTER.

Additionally, the energy integrals of COOP/COHP/COBI (i.e., ICOOP/ICOHP/ICOBI) are also helpful to analyze chemical bonds as they represent the number of electrons, bond strength measured by the contribution to the band‐structure energy, and bond order, respectively. Such information can also be automatically extracted by LOPOSTER from ICO*x*LIST.lobster (*x* = OP, HP, or BI) files and appended to the corresponding figures. In the NM situation, only one number will be provided whereas in the realm of magnetism, two numbers will be presented and denoted by *α/β* for spin‐majority/minority, respectively. By comparing the COOP/COHP/COBI data it is obvious that spin polarization eliminates antibonding levels in the very weak Ni–Ni bonds below the Fermi level (*E*_F_), in line with our previous study on MnNCN [27]. The choice of FM or AFM does not lead to significant changes in two‐center ICOOP/ICOHP/ICOBI for the Ni–Ni bond. Interestingly, upon defining an N–C–N three‐center bond for the [NCN]^2−^ entity, which is supposed to be electron precise, negative COBI^(3)^ levels and negative ICOBI^(3)^ values are observed, as shown in the very right column of Figure 2. As the orbital‐wise data have already been output by LOBSTER, we can take this advantage and directly proceed to check the orbital origin of negative COBI^(3)^ values and ICOBI^(3)^. The negative ICOBI^(3)^, indicating electron richness, can be mainly attributed to the three‐center interactions of p_*y*_–p_*y*_–p_*y*_ and p_*z*_–p_*z*_–p_*z*_ orbitals from atoms N4, C8, and N5, respectively, in addition to simple two‐center σ interactions between s and p_*x*_ orbitals. Therefore, we draw the (trivial) conclusion that there exist two delocalized *π* bonds in the [N=C=N]^2−^ entity defying the conventional bonding scheme where two‐center C–N *π* bonds are perpendicular to each other. On the contrary, there should exist three‐center four‐electron (3*c*–4*e*) interactions in the [NCN] entity. The reason leading to this phenomenon could stem from its symmetry. It is obvious that the [NCN] entity possesses the *D*_∞h_ point group. Therefore, the π_*y*_ and π_*z*_ orbitals need to be completely delocalized over the whole molecule to comply with the perfect *D*_∞h_ symmetry in the electron density. The negative COBI^(3)^ and ICOBI^(3)^ values of the [NCN] entity are the consequences of such delocalizing, electron‐rich features.

**FIGURE 3:**
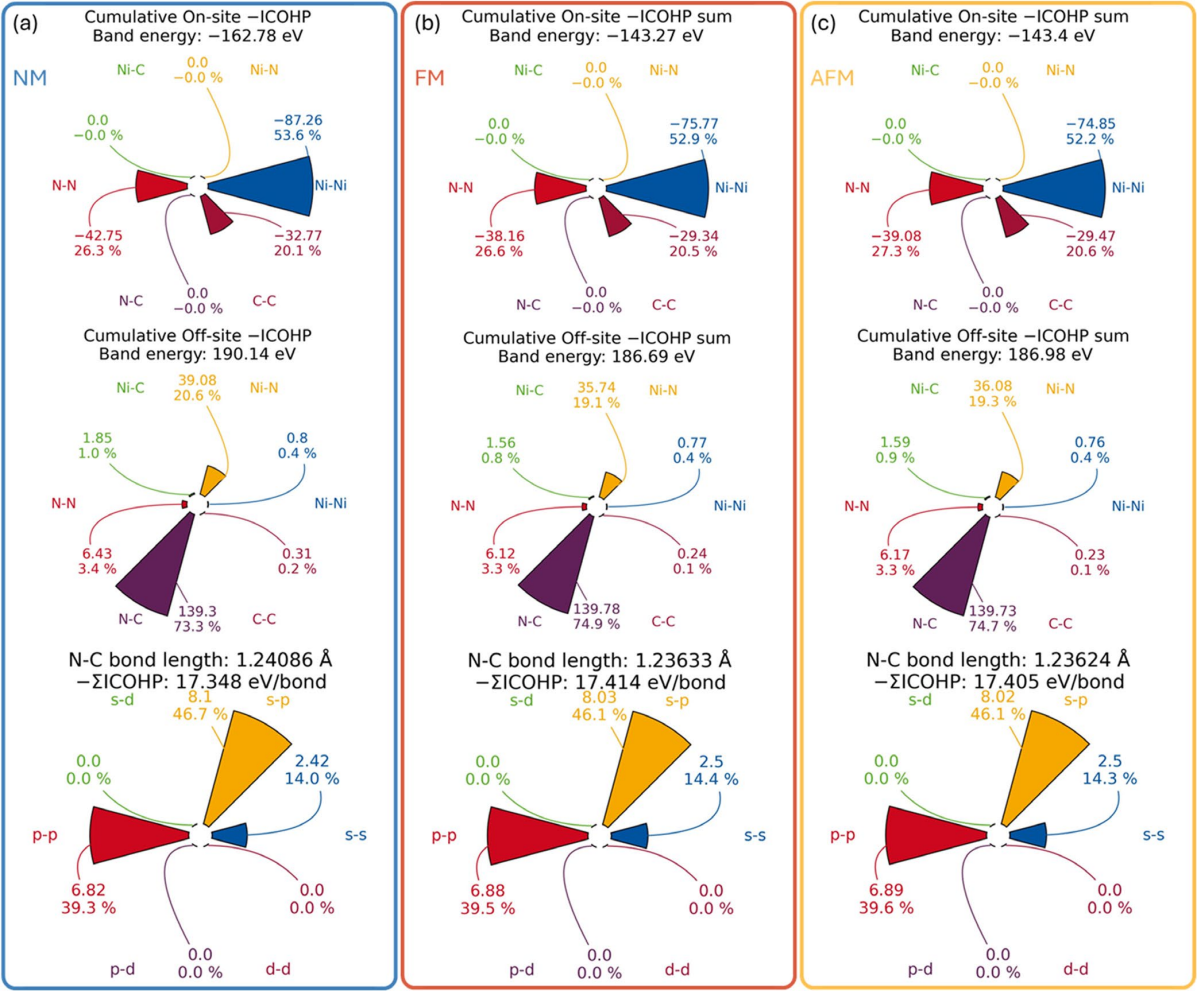
Cumulative ICOHP in (a) NM, (b) FM, and (c) AFM ground states of NiNCN, partitioned into various on‐site (top panel) and off‐site interactions (middle panel) and further partitioning of the C–N ICOHP data into orbital‐wise contributions.

More information regarding the integrals can be extracted by LOPOSTER to reveal the contribution of a certain pair of atoms/elements and compare it with the other interactions (dubbed cumulative ICO*x*, *x* = OP, HP, or BI), as shown in the upper and middle panels of Figure 3.

The on‐site part interactions are meaningful if and only if 0 is set as the lower bound in the lobsterin file. The true magnetic ground state correlates with the most positive –ICOHP values for Ni atoms, designating the weakest destabilizing effects stemming from the crystalline electric field (CEF). The middle panel of Figure 3 is needed to evaluate how one particular bonding (type) compares with all the others. It is clear that the strongest stabilizing effect in NiNCN originates from the C–N interactions inside [NCN]^2−^, accounting for ≈75% of the total stabilization contribution, showing the importance of the complex carbodiimide anion and its *internal* bonding. The less stabilizing effect from Ni–N interactions (≈20%) can also be detected, suggesting a rather strong indirect exchange interactions [20, 27]. In contrast, Ni–Ni interactions, as alluded to already, are rather weak compared with the other strong interactions, amounting to only ≈ 0.4% of stabilizing effects. This is not only because of antibonding levels below the *E*_F_, but also because of rather small absolute values of COHP, as shown in Figure 2, for the entire energy range below *E*_F_, mirroring the weak nature of Ni–Ni interactions as a function of the comparatively large interatomic distance. Within the most stabilizing bond (data shown in the bottom panel of Figure 3), it is found that the s–p interactions generating the σ bond account for almost half of the stabilizing effect for the C–N bonds. Comparing three configurations, we also find that the stability relies more on the C–N bond upon considering spin polarization, despite the fact that spin polarization should trivially affect the magnetically inactive atoms. This observation suggests a significant covalency decrease in the Ni–N interactions. The energy lowering by spin polarization decreases the –ICOHP values for Ni–N by ≈10% whereas the –ICOHP values for the C–N bond remain almost unchanged. Consequently, spin polarization makes the entire system more ionic. This is also supported by the increased Madelung energy, as tabulated in Table 1.

**TABLE 1:** Madelung energies (kJ mol^−1^) for NiNCN under various circumstances.

Method	Non‐magnetic	Ferromagnetic	Antiferromagnetic
Mulliken charge	−1516	−1666 (9.9%)	−1684 (11.0%)
Löwdin charge	−1034	−1180 (14.1%)	−1197 (15.8%)

*Note:* The changes with respect to the non‐magnetic situation are denoted in parentheses.

Regardless of the charge‐dependent method to calculate the Madelung energy of different ground states, we can always observe significant increases in Madelung energies for ferromagnetic and antiferromagnetic cases, compared with the non‐magnetic case. Please also note that for spin‐polarized states, the distance between the cation and anion *widens* due to the more diffuse feature of the spin minority (*β*) channel [28] which is also more covalently bonding.

Judging from the charge distribution, as summarized in Table 2, the emergence of magnetism is accompanied by a small additional charge transfer from Ni to the nonmetal atoms. That is to say that the ionicity of the Ni–N bonds does increase, despite the underlying covalency. Within the scope of magnetism, augmenting the existing findings [27], the choice of magnetic order shows its correlations with maximized Madelung energy (negative sign only dubs stabilizing effects) and largest ionicity for the Ni–N bonds. Summarizing the findings till now, we are, to a large extent, in a magnetic variant of situation resembling what was discussed by the principle of maximal hardness (PMH).

**TABLE 2:** Charge distribution (unit: *e*) in NiNCN regarding various magnetic ground states.

	Ni		C		N	
	Mulliken	Löwdin	Mulliken	Löwdin	Mulliken	Löwdin
Nonmagnetic	1.21	1.02	0.26	0.19	−0.74	−0.60
Ferromagnetic	1.24	1.06	0.33	0.25	−0.78	−0.66
Antiferromagnetic	1.25	1.07	0.32	0.25	−0.78	−0.66

### 3.3 LCFO Module

**FIGURE 4:**
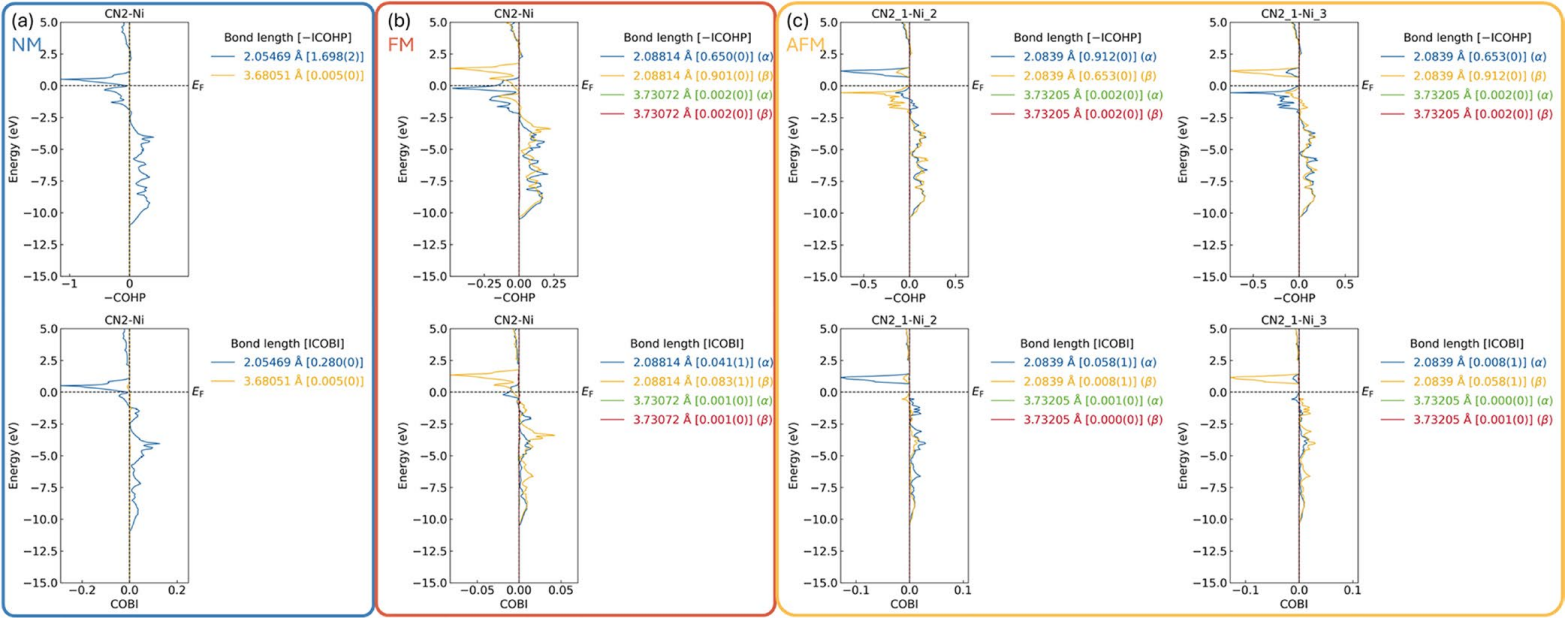
Interactions between fragment NCN and Ni atoms in cases of NM, FM, and AFM orderings, showing COHP (top) and COBI (bottom) data. In the legends, ICOHP/ICOBI values are also presented and colored the same as the corresponding curve.

Another appealing feature of LOBSTER is its recently implemented linear combination of fragment orbitals (LCFO) algorithm to derive such quantum‐chemical fragments also from plane‐wave results [24]. Thus far, to our knowledge, there is no ready‐to‐use postprocessor for such functionality. After manually defining the “molecule” fragment in the lobsterin file, i.e., the [NCN]^2−^ complex‐anion entity in our case, the interactions between atoms, between atoms and fragments, and between fragments can be quantitatively analyzed. Figure 4 presents an example of such analysis with an emphasis on CN_2_–Ni interactions, so we are looking at interactions between Ni and the *entire* [NCN]^2−^ complex anion. There are bonding/antibonding interactions at lower/higher energy below the Fermi level, and the ICOHP values for CN_2_–Ni slightly decrease as a consequence of emerging magnetic orderings.

Comparing FM and AFM models, the ICOHP values for CN_2_–Ni remain almost unchanged for the two magnetic orderings. However, ICOBI values, in stark contrast with ICOHP analogs, decrease by ≈56% from NM to FM and ≈76% from NM to AFM. This finding is consistent with our conclusions drawn in the AO module section. It is also worth noting that in the AFM setting, the CN_2_–Ni interactions become almost solely ionic in one of the spin channels, supporting the determinative role of ionicity in the choice of magnetic ordering.

**FIGURE 5:**
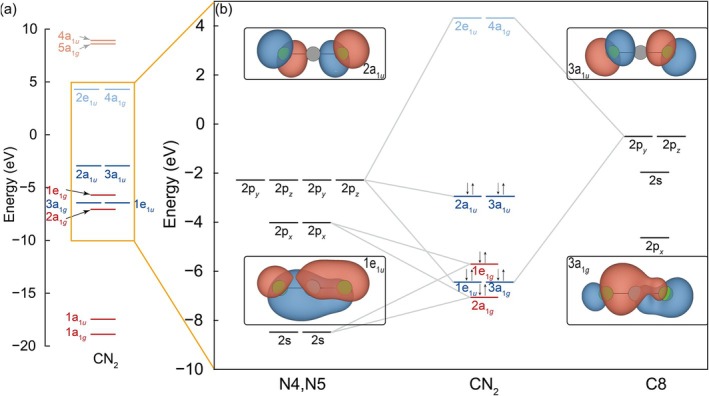
The MO diagrams of the [NCN] entity in NiNCN. All MOs are presented in (a) whereas a zoomed‐in diagram (b) focuses on those MOs formed by interactions of p orbitals. Occupied MOs are marked by a pair of antiparallel arrows and colored in pale colors. Singly and doubly degenerate MOs are colored in red and blue, respectively. On the right side, MOs are connected with the significantly contributing AOs. The insets present the MOs corresponding to the 3*c*–4*e* bonds. Please note that the Mulliken symbols assigned to the molecular orbitals are based on the best matching irreducible representation of the molecule's character table. Because the orbitals are distorted by the surrounding ligand field, this match is imperfect such that the *a* and *e* labels do *not* refer to whatever degeneracy.

After analyzing the interactions between a user‐defined fragment and its neighbors, it is time to examine the internal interactions *within* the [NCN]^2−^ group. The classical way to analyze is via the molecular orbital (MO) diagram, as sketched in Figure 5. All 12 MOs have been plotted in Figure 5a and a zoomed‐in perspective has been provided for better resolution of the MOs near *E*_F_. Judging from the MO diagram in Figure 5b, the electron‐rich feature (recapping the results from COBI^(3)^) of [NCN] entity can be unambiguously unraveled. The non‐bonding contribution from the central carbon atom and the slightly bonding contributions from the two p_*y*_/p_*z*_ orbitals in terminal nitrogen atoms lead to the populated MOs designated 2a_1*u*_ and 3a_1*u*_, respectively. Such a situation resembles what was discussed in phase‐change chalcogenides [29]. The difference is that, in contrast to the 3*c*–4*e* σ bonds previously discovered in those phase‐change materials, the multicenter bond discussed in NiNCN (a pseudo‐chalcogenide) is a 3*c*–4*e* π bond.

**FIGURE 6:**
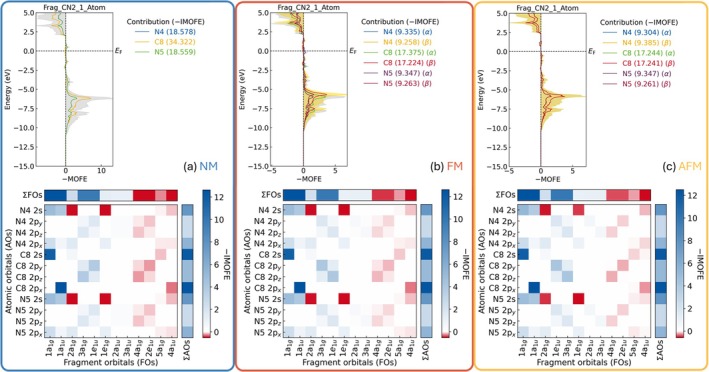
MOFE of the NCN group and matrix representation of IMOFE, considering (a) NM, (b) FM, and (c) AFM ground states. The matrix addresses both which collection of atomic orbitals forms a molecular orbital and how each atomic orbital contributes to the molecular orbital. The sign of IMOFE is revealed by different colors: Red denotes antibonding contributions, white denotes non‐bonding contribution, and blue denotes bonding contributions.

Yet another method to quantitatively evaluate the internal interactions apart from only looking at the C–N bonds is to compute the molecular orbital formation energy (MOFE) [24], an alternative partitioning scheme for the band energy and its integral (dubbed IMOFE), not purely in terms of atomic but, instead, of the combination going from atomic to molecular orbitals. From the MOFE course presented in Figure 6, we find that the position of Fermi level is rather optimized, essentially residing at the nonbonding regime. Interestingly, no significant stabilizing or destabilizing levels show up near the Fermi level, thereby corroborating the existence of a complex anion, a posteriori, whose electronic structure is nicely adjusted. As alluded to already, the IMOFE energy integrals can also offer a lot of valuable information. LOPOSTER automatically extracted those values from the IMOFELIST.lobster file and plotted them as a matrix, as shown in the bottom part of each panel in Figure 6. By doing so, it is quite clear to filter out the most prevailing contribution of atomic orbitals to a molecular orbital, be it bonding or antibonding. To facilitate further analysis, summations were performed along different directions of the IMOFE matrix to unveil the overall contribution of each atomic orbital or fragment orbital to the stability of the fragment, the results of which have been visualized in the upper and right sides of corresponding IMOFE matrix and termed as ΣFOs and ΣAOs, respectively.

As regards atomic orbitals, the 2 s and 2 p_*x*_ orbitals from the central carbon atom are the most stabilizing components. As regards the fragment orbitals, from lower energy levels to higher energy ones, the fragment orbital changes from stabilizing via nonbonding to destabilizing, consistent with the results shown in the upper panel of Figure 6. Additionally, those features hold throughout, regardless of magnetic ordering, justifying the complex anion and pseudo‐molecular feature of [NCN]^2−^ entity in NiNCN.

### 3.4 Kspace Module

**FIGURE 7:**
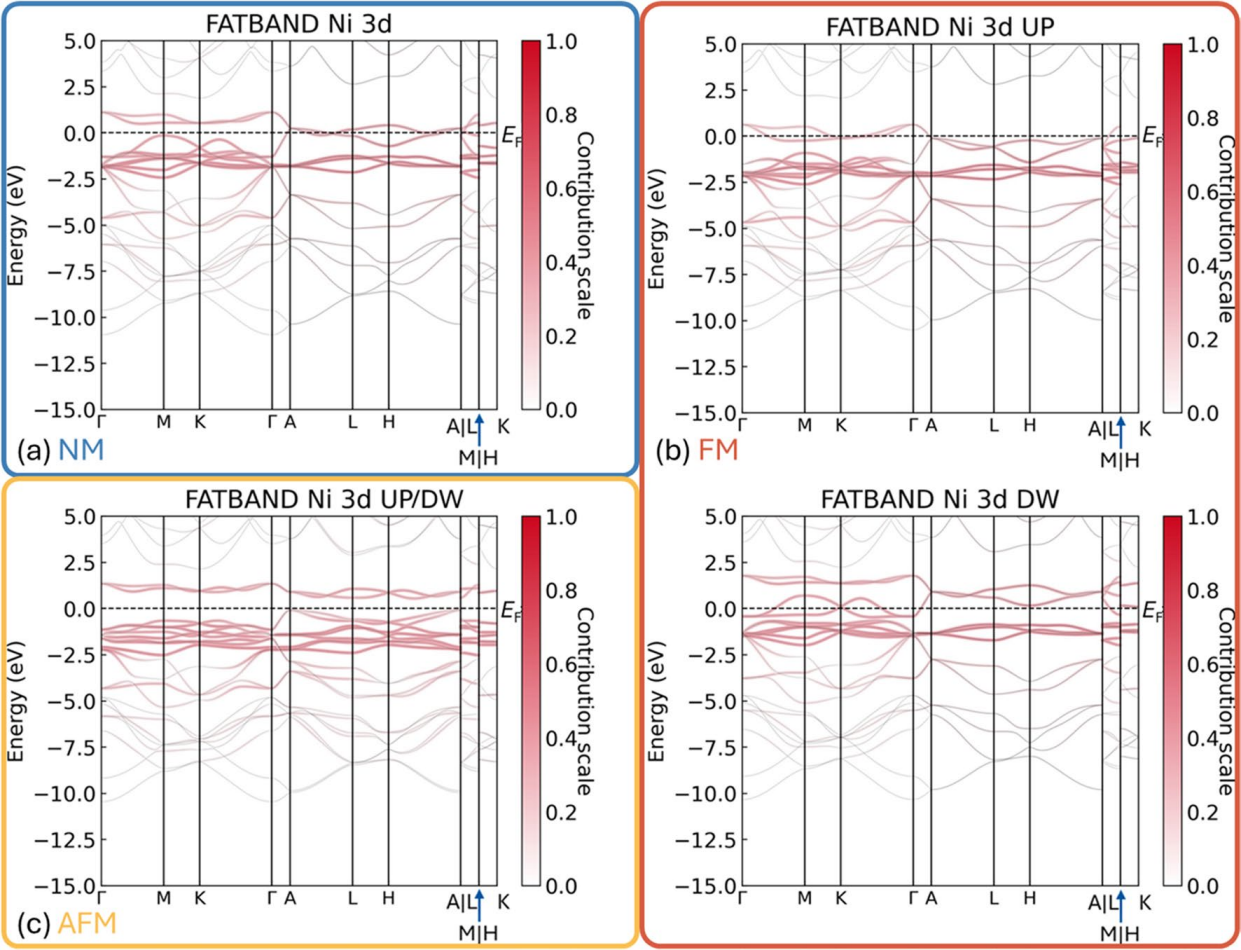
Fatband data for Ni′s 3d orbitals in NiNCN considering (a) NM, (b) FM, and (c) AFM ground states. Eigenvalues have been colored according to the contributions from Ni′s 3d orbitals. White is used to show no contribution (contribution scale is 0) whereas red represents full contribution (contribution scale is 1).

After harvesting abundant information from real space, it is time to navigate in reciprocal space helped by LOPOSTER. LOBSTER has functionalities to perform fatband and *k*‐space COHP analyses. To the knowledge of the authors, python packages such as pymatgen [12], lobsterpy [13] and PyProcar [30, 31] can process and plot the fatband data from LOBSTER whereas there is still no available code for *k*‐space COHP data, but algorithms to process and plot both fatband and *k*‐space COHP data have been implemented in LOPOSTER. To perform those analyses, a separate calculation must be executed as additional *k*‐points have to be appended to the previous SCF calculations. LOPOSTER helps to create a separate folder and copy necessary files into it, followed by modifying the lobsterin file and appending more *k*‐points to the file KPOINTS (currently only supporting VASP); please refer to LOBSTER's user guide for more details. After performing the additional calculations, the data generated by LOBSTER will be utilized as input files for LOPOSTER. It identifies if the preceding LOBSTER run was a *k*‐space calculation and automatically invokes the LOPOSTER, reads all necessary results, reformats the eigenvalues at each *k*‐point into a matrix containing information on each band, and plots the matrix with weights given by the contribution from a certain atom/orbital. For illustration, Figure 7 presents the fatband results of NiNCN, focusing on the 3d orbitals of the Ni atoms. These 3d orbitals mainly contribute to the energy range between −2.5 and 2 eV in all three cases, coinciding with our expectations of energy ranking for 3d orbitals in Ni. The fact that the trajectories of energy bands regarding Ni's 3d orbitals are rather flat along the high symmetry path also reproduces the insignificant overlap of those orbitals with their neighboring orbitals.

**FIGURE 8:**
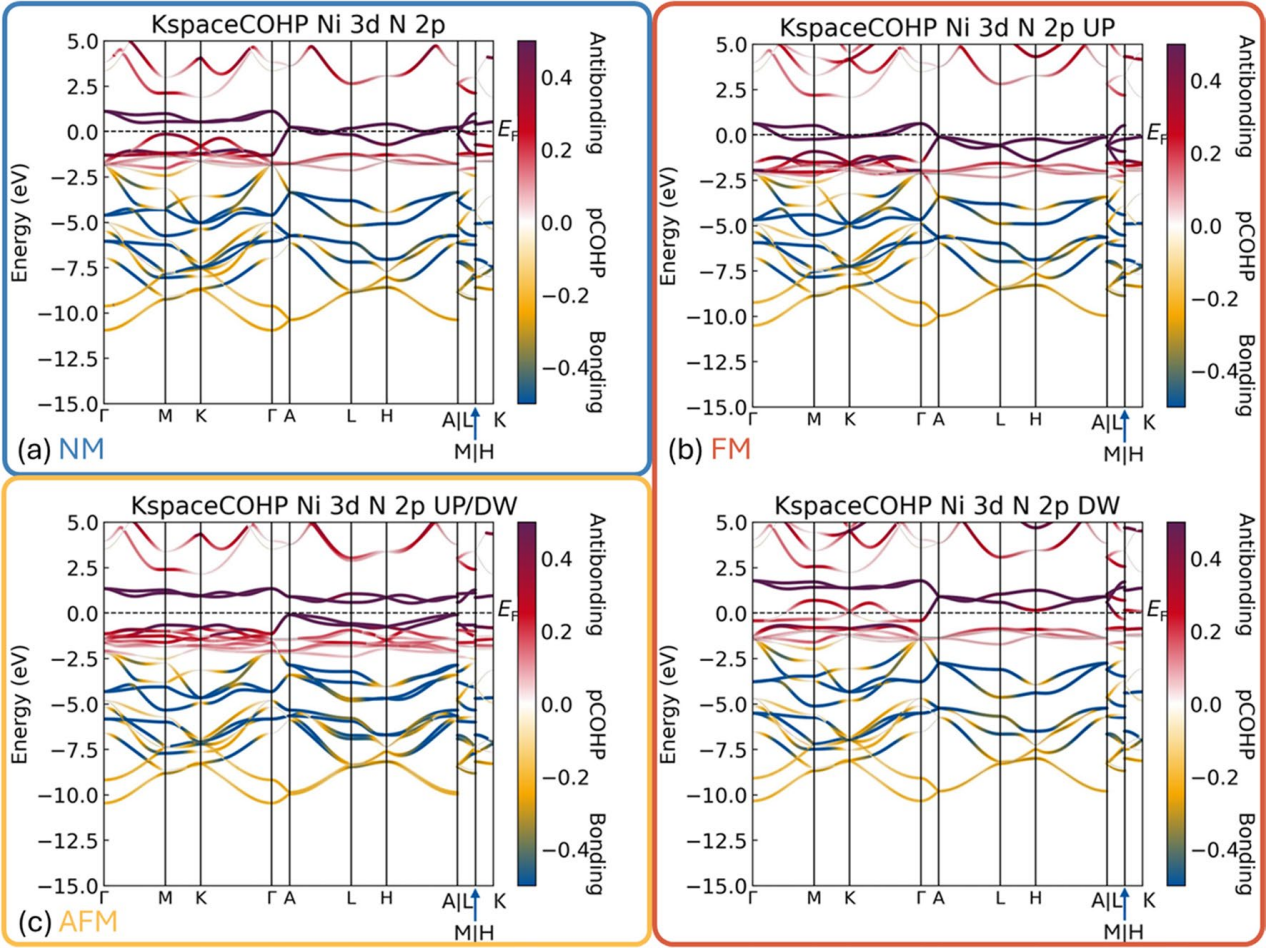
*k*‐space COHP data between Ni′s 3d orbitals and N's 2p orbitals in NiNCN, in cases of (a) NM, (b) FM, and (c) AFM. Eigenvalues are colored according to the corresponding COHP values. Blue and yellow are used for bonding, red and violet are used for antibonding, and white is used for nonbonding interactions, respectively.

Similarly, LOPOSTER automatically finds LOBSTER's *k*‐space COHP data and reformats the data to construct a three‐dimensional tensor whose three directions are *k*‐points, energies, and pair‐wise interactions, respectively. Subsequently, slices of the constructed matrix will be made to calculate the desired data for plotting. If necessary, the same orbitals (i.e., same principle, azimuthal, and magnetic quantum numbers) from the same element in the system will be summed up. Alternatively, data from orbitals with the same principal and azimuthal quantum numbers will be put together to gain some macroscopic information. The *k*‐space COHP data processed by LOPOSTER have been depicted in Figure 8.

The dominating interactions arise between the 3d orbitals from Ni and the 2p orbitals from the coordinating N atoms. We are particularly interested in those interactions, so‐called p–d mixing (or hybridization, physics language) interactions, as they are usually considered to be the source of metal‐nonmetal stabilization. That being said, our findings from *k*‐space COHP allow a more energy‐resolved analysis, augmenting previous research which usually attributes the stability to the p–d interactions because their energies are in the vicinity of the Fermi level (*E*_F_). Recalling Figure 3, however, as regards total and band‐structure energy, the strongest stabilizing effects originate from the C–N interactions inside the complex anion, their energies being *far* below *E*_F_, with the Ni–N interactions being the second strongest.

Let's also examine the roles of p–d interactions between Ni and N. Independent of the magnetic ordering, the change of *k*‐space COHP follows the same tendency: going down from *E*_F_, the *k*‐space COHP values undergo a transition from strong antibonding (above −2.5 eV) to strong bonding. This translates to a mainly destabilizing effect from the *E*_F_ down to about −2.5 eV and a mainly stabilizing effect from −2.5 eV down. Tracking the trajectory of the same band along the high symmetry *k* path, the stabilizing effect is also slightly *k*‐dependent. Even though the overall Ni–N interaction is net bonding (judged from ICOHP value in Figure 3), eigenvalues along the high symmetry path encompass bonding, nonbonding, and also antibonding effects, as given by the coloring to indicate interactions between Ni′s 3d orbitals and N's 2p orbitals. That being said, the net sum of those values agrees well with the data postprocessed by the AO module, displaying bonding levels at energies far below the *E*_F_ and antibonding levels in the vicinity of *E*_F_. Therefore, the effects of p–d interactions do not simply increase the stability of the system as those effects are both energy‐ and *k*‐dependent. We would hardly rely on the local features of DOS or contributions from atomic orbitals near *E*_F_ to reveal the effects of those levels on the stability of the system as the DOS taken alone does not contain the phase information.

### 3.5 Performance of LOPOSTER

The LOPOSTER code has been written to make postprocessing simpler, not more complicated, so it is worth discussing its performance. Unlike the other methods articulated above, it is not necessary to know which bond(s) to analyze. Almost all the chemical bonding information regarding a given system would be exported by LOBSTER, and the postprocessing time needed by LOPOSTER is marginal in most cases if compared with the time consumption by LOBSTER. Table 3 tabulates the timings of LOBSTER and LOPOSTER in different cases. It is found that the *additional* time needed by LOPOSTER accounts only 0.5%–0.7% and 2%–5% of the original LOBSTER time if atomic‐orbital (AO) and fragment‐orbital (LCFO) is required. Only if *k*‐space analysis is required, LOPOSTER demands an additional 46%–90% time. In the present case, the numbers of interactions processed by LOPOSTER are 16,101 and 28,602 for non‐magnetic and magnetic cases, respectively. The fact that such a huge number of interactions can be postprocessed within 1.5 min (on an Intel Xeon E5‐2680 v4 CPU with 28 cores and 2.40GHz base frequency) manifests the high efficiency of algorithms implemented in LOPOSTER and the great potential of such code to be integrated into high‐throughput chemical bonding analysis.

**TABLE 3:** Comparison of time (s) consumed on LOBSTER and LOPOSTER runs.

	Real space: AO		Real space: LCFO		Kspace	
	LOBSTER	LOPOSTER	LOBSTER	LOPOSTER	LOBSTER	LOPOSTER
NM	4444.46	34.93	780.02	14.60	110.56	50.83
FM	13962.33	70.38	340.88	17.49	213.24	191.48
AFM	13749.42	67.76	342.37	17.74	224.76	164.14

## 4 Conclusions

In summary, we have developed LOPOSTER, a robust and highly efficient algorithm for fully automated postprocessing of chemical bonding analysis as produced by LOBSTER. Although the python script itself can be directly executed, we would still like to distribute the prepacked standalone executable files for all kinds of operating systems. This would be helpful for researchers without any experience in Python scripts. By doing so, programming skills are not required for performing chemical bonding analysis in a high‐throughput manner. As an example, NiNCN was chosen to showcase the functionalities of LOPOSTER. Analyses of its chemical bonding information were carried out both in real and reciprocal space. In real space and using localized atomic orbitals, the emergence of magnetic ordering and the choice of the magnetic ground state is not only related to the depletion of antibonding levels in the vicinity of *E*_F_, but also leads to enhanced ionicity in the system, coinciding with the principle of maximal hardness. The magnetic ground state guarantees the maximized ionicity in the system. On on‐site and off‐site cumulative ICOHP analyses, as visualized by LOPOSTER, easily provides an overview of chemical bonds in the entire system and clearly suggests the most stabilizing interactions for such a system. Such analysis can be performed by LOPOSTER to further reveal the orbital‐wise origin of stabilizing interactions. Additionally, after adopting the LCFO algorithm and retrieving the results using molecular orbital basis, the information of interactions between cations and complex anions is accessible. Based on this, we could find out that, appending to the existing results that focused on the Mn–N bond [27], the interactions of Ni–[NCN] clearly reveal the enhanced ionicity after the emergence of magnetic ordering as the ground state. In addition, the results of interactions within the [NCN]^2−^ entity can also be visualized by means of both trajectories of MOFE curves and IMOFE matrix. LOPOSTER can also be utilized to visualize the results in reciprocal space. Fatband and *k*‐space COHP data in NiNCN are processed with marginal addition of computational costs (< 1.5 min). With proper settings, all the results can be processed by only a single LOPOSTER execution.

## Conflicts of Interest

The authors declare no conflicts of interest.

## Data Availability

The LOPOSTER code is available as a standalone executable file via the GitHub link https://github.com/WangYiXu92/LOPOSTER. It will also be provided on the download page of http://cohp.de/.
